# Influence of Nanosized CoTiO_3_ Synthesized via a Solid-State Method on the Hydrogen Storage Behavior of MgH_2_

**DOI:** 10.3390/nano12173043

**Published:** 2022-09-01

**Authors:** Nurul Amirah Ali, Muhammad Syarifuddin Yahya, Noratiqah Sazelee, Muhamad Faiz Md Din, Mohammad Ismail

**Affiliations:** 1 Energy Storage Research Group, Faculty of Ocean Engineering Technology and Informatics, University Malaysia Terengganu, Kuala Nerus 21030, Malaysia; 2 Department of Electrical and Electronic Engineering, Faculty of Engineering, National Defence University of Malaysia, Kem Sungai Besi, Kuala Lumpur 57000, Malaysia

**Keywords:** MgH_2_, hydrogen storage, catalytic activity, CoTiO_3_

## Abstract

Magnesium hydride (MgH_2_) has received outstanding attention as a safe and efficient material to store hydrogen because of its 7.6 wt.% hydrogen content and excellent reversibility. Nevertheless, the application of MgH_2_ is obstructed by its unfavorable thermodynamic stability and sluggish sorption kinetic. To overcome these drawbacks, ball milling MgH_2_ is vital in reducing the particle size that contribute to the reduction of the decomposition temperature. However, the milling process would become inefficient in reducing particle sizes when equilibrium between cold-welding and fracturing is achieved. Therefore, to further ameliorate the performance of MgH_2_, nanosized cobalt titanate (CoTiO_3_) has been synthesized using a solid-state method and was introduced to the MgH_2_ system. The different weight percentages of CoTiO_3_ were doped to the MgH_2_ system, and their catalytic function on the performance of MgH_2_ was scrutinized in this study. The MgH_2_ + 10 wt.% CoTiO_3_ composite presents the most outstanding performance, where the initial decomposition temperature of MgH_2_ can be downshifted to 275 °C. Moreover, the MgH_2_ + 10 wt.% CoTiO_3_ absorbed 6.4 wt.% H_2_ at low temperature (200 °C) in only 10 min and rapidly releases 2.3 wt.% H_2_ in the first 10 min, demonstrating a 23-times-faster desorption rate than as-milled MgH_2_ at 300 °C. The desorption activation energy of the 10 wt.% CoTiO_3_-doped MgH_2_ sample was dramatically lowered by 30.4 kJ/mol compared to undoped MgH_2_. The enhanced performance of the MgH_2_–CoTiO_3_ system is believed to be due to the in situ formation of MgTiO_3_, CoMg_2_, CoTi_2_, and MgO during the heating process, which offer a notable impact on the behavior of MgH_2_.

## 1. Introduction

In recent decades, hydrogen has emerged as the most viable energy carrier, especially in the transportation sector [1]. As far as we are concerned, the usage of fossil fuels has led to a global energy crisis and environmental pollution [2]. Thus, energy transition from fossil fuel-based to renewable energy is required. As environmentally-friendly energy storage, the best solution is to convert energy from “green” sources into chemical storage [3]. Hydrogen has received outstanding interest as an energy carrier because it has a high energy content (142 MJ/kg) [4]. Hydrogen might supersede natural gas and solid fuels as an energy source by 2050. It is anticipated that it will be extensively used in the transportation, chemical, and long-term aviation and maritime sectors [5]. The use of hydrogen as an energy carrier will ultimately aid humankind in achieving a “low” or “zero” carbon future. Recently, various countries have started using hydrogen energy, particularly in transportation. For instance, the Hyundai Nexo hydrogen fuel cell vehicle (FCV) sold 1000 units in South Korea in 2019 [6], and the company is also setting up a number of hydrogen refueling stations [7]. Nevertheless, hydrogen-based energy requires a convenient and a reliable method of storing energy. How to store hydrogen is a vital phase for delivering a stock of hydrogen fuel to consumers for energy storage, fuel cell vehicles, stationary applications, and portable devices.

Commonly, hydrogen can be stored in three different forms, namely, (i) gas, (ii) liquid, and (iii) solid-state. The current hydrogen storage method for compressing gas requires high pressure, which is in the range of 350–700 bar H_2_ at room temperature, whereas liquid-form storage needs low temperature for the operation (−253 °C) [8,9]. The solid-state form appears to be the most effective technique compared to gas and liquid hydrogen because it has several benefits, including safety, high hydrogen capacity, modest working pressures, and excellent energy efficiency [10,11,12]. Throughout the last several decades, various types of solid-state material have been discovered. The solid-state approach of storing hydrogen has two techniques, namely, chemisorption and physisorption [13]. As a hydrogen storage technique, chemisorption in metal hydrides has drawn considerable attention. The advantage of this approach is that it can store a large amount of hydrogen in a small volume [14].

Among the various solid-state materials explored for hydrogen storage, MgH_2_ has been regarded as a viable option to store hydrogen for onboard applications on the strength of its high hydrogen capacity (7.6 wt.%), high energy density (9 MJ/kg) over all the reversible hydrides, and outstanding reversibility [15]. However, the obstacles of MgH_2_ for practical application include high temperature (>400 °C) to release hydrogen, sluggish sorption kinetics, and very stable thermodynamic properties (ΔH = 76 kJ/mol H_2_) [16,17]. To tackle these drawbacks, several efforts, such as nanostructuring, nanoconfinement, alloying, and the addition of catalyst [18,19,20,21] have been proposed and developed to modify the Mg-based system performances. In this study, the center of interest is to reduce the decomposition temperature and improve the kinetics performance of MgH_2_ by adding catalysts. Adding metal oxide [22,23,24], metal [25,26], metal halides [27], carbon material [28,29], and nanosized alloys [30,31] to MgH_2_ as catalysts have been highlighted as notable approaches to boost its hydrogen storage performance.

Among the catalysts, metal oxide has attracted growing interest because of its profound influence on the behavior of MgH_2_. Moreover, transition metals and their compounds have been proven to ease hydrogen dissociation and recombination [32]. Transition metals and their oxides are able to provide more sites for Mg and MgH_2_ nucleation, which ameliorates the hydrogen sorption kinetics [33]. Moreover, these transition metal oxide are easily reduced and converted into low-valence transition metal oxide or hydride due to the high reductivity of Mg/MgH_2_, which has been identified as a promising catalyst for dehydrogenation of MgH_2_ [34]. This is due to the significant interaction between the s-states of H and the d-states of low-valence transition metals, which could weaken the Mg-H bonds of MgH_2_ and enhance its reversible hydrogen storage performance. As reported by the earliest study, doping Ti, Mn, V, Fe, and Ni presents a rapid desorption process of MgH_2_ [35]. Thereafter, another series of transition metals, Co, Cu, and Nb, display remarkable catalytic function on the hydrogen release of MgH_2_ [36,37]. It is predicted that the oxides of transition metals proffer efficient catalytic activity on the performance of MgH_2_. For instance, adding TiO_2_ could speed up the absorption and desorption process of MgH_2_, enable the absorption of 5.3 wt.% H_2_ in only 44 s, and release 6.4 wt.% H_2_ in 700 s. Moreover, Yan et al. [38] demonstrated that adding NiTiO_3_ could lower the temperature for the release of hydrogen as well as the activation energy of MgH_2_ and result in faster hydrogen desorption and absorption. Furthermore, Shan et al. [39] reported that the presence of CoFe_2_O_4_ could speed up the hydrogen dehydriding of MgH_2_. The composite sample of MgH_2_ + 9 mol CoFe_2_O_4_ began to decompose at 150 °C, which was lower than undoped MgH_2_ (340 °C), and it is capable of desorbing 6.11 wt.% H_2_. Then, Ismail et al. [40] conducted a further study on transition metal oxide. The addition of CuFe_2_O_4_ could reduce the temperature for release hydrogen from 340 °C to 250 °C. They concluded that the enhancement of the MgH_2_ behavior was due to the formation of MgO, Fe and Mg–Cu alloy that accelerates the hydrogen desorption of MgH_2_. Therefore, transition metal oxide catalysts are favorable in ameliorating the hydrogen storage behavior of MgH_2_ by boosting the electron transfer between Mg and H [32].

In this study, it is of interest to examine the catalytic activity of another transition metal oxide, CoTiO_3_. What is noteworthy, is that the catalytic efficiency is influenced by the dispersion of catalytic activity on the system. Nanocatalysts offer noticeably more active catalytic sites and intimate interaction because of their increased surface area. Thus, in this study, we synthesized nanosized CoTiO_3_ via a solid-state method. The addition of CoTiO_3_ was projected to remarkably boost the behavior of MgH_2_ by lowering the initial decomposition temperature and rapid hydrogen absorption/desorption kinetics. Although there is a previous study reported on the effect of CoTiO_3_ [41], there remains a lack of mechanism of interaction between MgH_2_ and CoTiO_3_. Furthermore, different synthesis methods present different catalytic activities. This study broadens the way to explore the effect of CoTiO_3_ and demonstrates the different catalytic activities of CoTiO_3_ on the hydrogen storage behavior of MgH_2_. Hence, in this paper, the influence of different weight percentages of CoTiO_3_ was systematically explored.

## 2. Materials and Methods

MgH_2_ (95% pure), Co_3_O_4_ (99.99% pure), anatase TiO_2_ (99.95% pure), and citric acid monohydrate (>98% pure) provided by Sigma Aldrich, St. Louis, MO, United States were used without any pretreatment. The nanosized CoTiO_3_ was synthesized using the solid-state method. The stoichiometric amount of Co_3_O_4_ and TiO_2_ were ground together for 15 min. Thereafter, citric acid monohydrate was added and continuously ground for 15 min. The powder was then calcined at 850 °C for 5 h. Thereafter, different weight percentages of CoTiO_3_ (5, 10, 15, and 20 wt.%) were added with MgH_2_ to study its catalytic activity on the hydrogen storage behavior of MgH_2_. The mixture was milled together in a planetary ball mill (NQM-0.4) at 400 rpm for 1 h.

Sievert-type pressure-composition-temperature apparatus from Advanced Materials Corporation, Pittsburgh, PA, United States which is also known as Gas Reaction Controller (GRC) apparatus, was used to conduct the temperature-programmed desorption (TPD) measurement and the hydrogen absorption and desorption experiment. The GRC provides a reliable and convenient way to evaluate desorption/absorption characteristics of materials. Temperature, capacity, and time data obtained through TPD measurements were used to determine the capacity, kinetics, and thermodynamic properties of material. The TPD measurement was conducted in a vacuum chamber heated to 450 °C (heating rate: 5 °C /min). For the isothermal tests, experiments were conducted at 200 °C, 33.0 atm hydrogen pressure (absorption kinetics), and 300 °C, 1.0 atm hydrogen pressure (desorption kinetics).

To study the thermal properties and to calculate the activation energy, differential scanning calorimetry (DSC, Mettler Toledo, Columbus, OH, United States)(DSC/TGA 1) was used. Six to eight milligrams of the sample were placed in a crucible and heated to 500 °C under an argon flow of 50 mL/min at four different heating rates (15 °C/min, 20 °C/min, 25 °C/min, and 30 °C/min). Rigaku MiniFlex, Tokyo, Japan, X-ray diffraction (XRD) with Cu Kα radiation was used to examine the phase structure. The patterns were scanned over diffraction angles from 20° to 80° at 2.00°/min. The sample microstructure and morphology were examined using a scanning electron microscope (SEM; JEOL, Akishima, Tokyo, Japan) (JSM-6350LA). The particle size distributions of the samples were calculated using Image J software. The SEM images was used to calculate the distributions size of the sample, where a number of measurements were collected for the diameter of the particle. Fourier transform infrared (FTIR) spectrometry was conducted in the range of 400–2000 cm^−1^ using IR Tracer-100, Shimadzu, Kyoto, Japan and Raman spectra were conducted at room temperature (0.1% power laser) using Renishaw Raman spectroscopy (532 nm radiation).

## 3. Results and Discussion

### 3.1. Synthesis of CoTiO_3_

**Figure 1:**
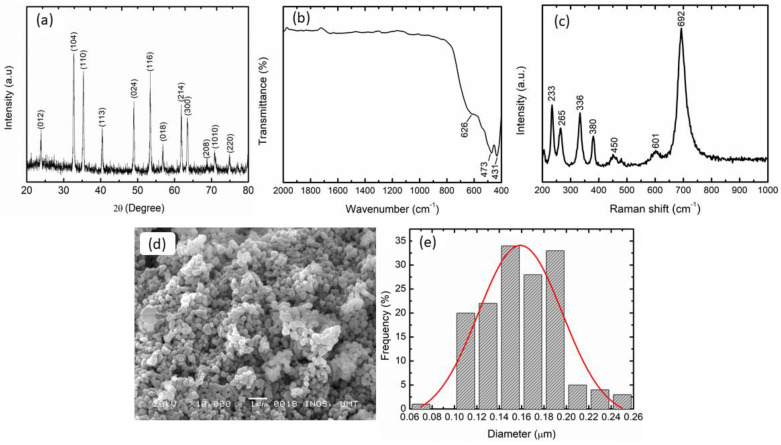
(**a**) XRD profile, (**b**) FTIR spectra, (**c**) Raman spectra, (**d**) SEM image, and (**e**) particle size distribution of as-synthesized CoTiO_3_.

The phase composition of the as-synthesized CoTiO_3_ was investigated using XRD, as presented in Figure 1a. The diffraction peaks at 2θ of 23.9°, 32.7°, 35.3°, 40.5°, 48.9°, 53.4°, 56.8°, 61.8°, 63.5°, 68.7°, 70.9°, and 74.8° correspond to the crystallographic plane of (012), (104), (110), (113), (024), (116), (018), (214), (300), (208), (1010), and (220). Evidently, all the peaks matched well with the spinel structure of CoTiO_3_ (JCPDS 15-866). No other peak was detected, confirming the formation of pure CoTiO_3_. The average crystallite size of the CoTiO_3_ was approximately 28.7 nm, calculated using the Scherrer formula (Equation (1)), as follows [42]:

L = Kλ/β cos θ(1)

where L, K, λ, β, and θ are referring to the average crystallite size, Scherer constant (0.94), X-ray wavelength, full width at half maximum, and diffraction angle, respectively. The FTIR spectrum of the as-synthesized CoTiO_3_ is presented in Figure 1b. The bands between 400 and 800 cm^−1^ are the typical peaks of CoTiO_3_ that correspond to the stretching vibrations of the metal ions [43]. The FTIR spectrum exhibited bands between 640 and 450 cm^−1^, which is due to Ti–O–Ti and Co–Ti–O band formation [44]. The strong band at around 431 cm^−1^ corresponds to the bond of Co–O [45]. Maensiri et al. [46] and Rashad et al. [47] stated that the peaks in the range of 450–600 cm^−1^ were ascribed to the Ti–O–Ti bond. Thus, the peaks at 473 cm^−1^ are assigned to the Ti–O–Ti bond [48] and the peaks at 626 cm^−1^ correspond to the Ti–O–O bond [46]. Moreover, the Raman spectra in Figure 1c confirm the formation of CoTiO_3_ [44]. The strongest Raman modes observed around 692 cm^−1^ were attributed to the high frequency of the vibrational mode of the CoO_6_ octahedra, known as the symmetry stretching mode, while other remaining Raman modes correspond to the lattice vibrations of the phonon modes [43]. The SEM image (Figure 1d) of the as-synthesized CoTiO_3_ revealed that the as-synthesized nanosized CoTiO_3_ comprises a good dispersion of eclipse-like grain shape. The particle size of the as-synthesized CoTiO_3_ is measured using Image J software, with an average particle size of 0.16 µm. The distributions of the particle sizes are depicted in a histogram in Figure 1e. The results show that it is relevant to conclude that pure nanosized CoTiO_3_ was successfully synthesized using the solid-state method.

### 3.2. Hydrogen Storage Properties

**Figure 2:**
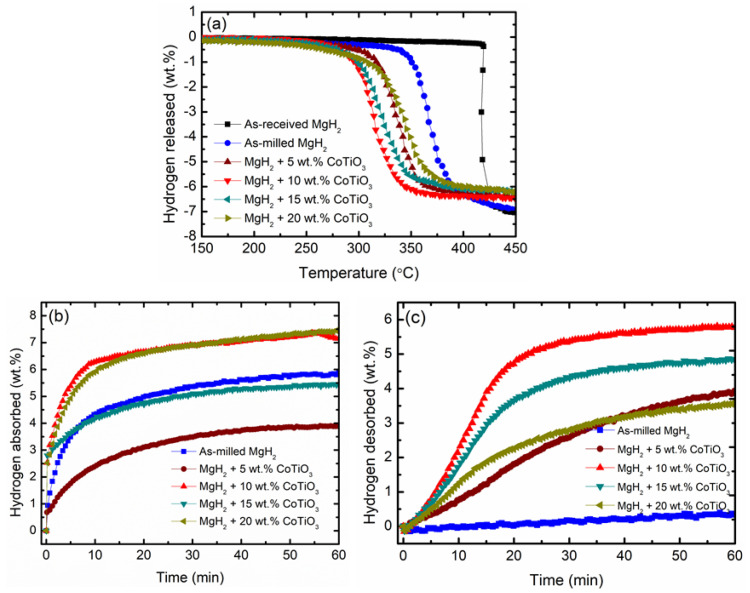
(**a**) TPD, (**b**) hydrogen absorption at 200 °C under 33.0 atm H_2_ pressure, and (**c**) hydrogen desorption curve at 300 °C under 1.0 atm H_2_ pressure of the MgH_2_–CoTiO_3_ system.

The catalytic activity of the as-prepared CoTiO_3_ on MgH_2_ was studied by TPD experiments, as depicted in Figure 2a. The performance of the modified MgH_2_ with CoTiO_3_ was significantly enhanced compared to undoped MgH_2_. In terms of decomposition temperature, commercial MgH_2_ started decomposing at 420 °C, whereas as-milled MgH_2_ started at approximately 340 °C. This outcome implies that the process of ball milling for 1 h is beneficial in lowering the decomposition temperature of MgH_2_ as it decreased by 80 °C when compared with commercial MgH_2_. This result is consistent with a prior study that found that enhanced performance of metal/complex hydride is due to the milling process, which is effective in refining the particle size [49]. The milling process in essential in reducing the particle size, which contributes to the decrease of the decomposition temperature. However, the milling process would become inefficient in reducing particle sizes when the equilibrium between cold-welding and fracturing is achieved [50]. A previous study also reported that increasing the milling time causes the decomposition temperature to shift to higher temperatures due to the agglomeration of particles and reduction of their free surface area [51]. After augmenting MgH_2_ with 5 wt.% of CoTiO_3_, the onset decomposition temperature downshifted to 298 °C. The reduction in the decomposition temperature was more significant when the amount of CoTiO_3_ was increased to 10 wt.%. The MgH_2_ + 10 wt.% CoTiO_3_ sample began to decompose at 275 °C. For the 15 wt.% CoTiO_3_-doped MgH_2_, the decomposition temperature was almost similar to the MgH_2_ + 10 wt.% CoTiO_3_. The influence of different wt.% on the performance of MgH_2_ was further explored with 20 wt.% of CoTiO_3_. The composite sample of MgH_2_ + 20 wt.% CoTiO_3_ also began to release hydrogen lower than undoped MgH_2_, which started to release at 295 °C. However, the onset decomposition for the MgH_2_ + 20 wt.% CoTiO_3_ was slightly higher than MgH_2_ + 10 wt.% CoTiO_3_. This outcome was comparable with a previous study that found that the decomposition temperature for the 15 wt.% and 20 wt.% SrFe_12_O_19_-doped MgH_2_ was slightly higher than MgH_2_ + 10 wt.% SrFe_12_O_19_ [52]. Sulaiman et al. [53] also claimed the same condition, where the decomposition temperature for the MgH_2_ + 50 wt.% Na_3_FeF_6_ was slightly higher than for MgH_2_ + 10 wt.% Na_3_FeF_6_ and MgH_2_ + 20 wt.% Na_3_FeF_6_. This condition may be due to the excessive catalyst that may block the diffusion path of hydrogen, which limits the hydrogen diffusion [54]. Even the temperature for the hydrogen release for the 20 wt.% of catalyst was higher than that for the 10 wt.%; the temperature for the MgH_2_ to release hydrogen doped with a different weight percentage of CoTiO_3_ was significantly reduced when compared with undoped MgH_2_. Of note is the fact that the presence of CoTiO_3_ could modify the performance of MgH_2_ and be beneficial in lowering the decomposition temperature of MgH_2_.

The reversibility of the MgH_2_–CoTiO_3_ system was further studied via an absorption kinetic experiment conducted at a low temperature (200 °C). The doped sample was studied with a different weight percentage of CoTiO_3_, as demonstrated in Figure 2b. Among the four different wt.%, MgH_2_ + 10 wt.% CoTiO_3_ produces the fastest absorption rate and represents improved absorption kinetics performance than does undoped MgH_2_. As shown in Figure 2b, in the first 10 min, the as-milled MgH_2_ absorbed 4.3 wt.% H_2_, whereas the MgH_2_ + 10 wt.% CoTiO_3_ sample absorbed 6.4 wt.% H_2_. Even after completing the 60 min duration, the as-milled MgH_2_ could not attain as high capacity as that of the MgH_2_-doped 10 wt.% CoTiO_3_. In the meantime, the hydrogen absorbed for the 5, 15, and 20 wt.% of catalyst were 2.5, 4.1, and 6.0 wt.% H_2_, respectively, in the first 10 min. For the 5 wt.% of CoTiO_3_, the MgH_2_ + 5 wt.% CoTiO_3_ shows faster kinetics than milled MgH_2_ in the first 11s and intercept with the MgH_2_ at 12s and become slower than undoped MgH_2_. It can be concluded that the hydrogen absorption behavior was affected by the amount of catalyst. In the context of desorption kinetics, as depicted in Figure 2c, the MgH_2_-doped CoTiO_3_ composite displays a rapid desorption rate compared to undoped MgH_2_. Similar to the performance of the absorption kinetics, the MgH_2_ + 10 wt.% CoTiO_3_ presents a faster desorption kinetics rate compared to the 5, 15, and 20 wt.% CoTiO_3_-doped MgH_2_ samples. In the first 10 min, the undoped MgH_2_ roughly desorb hydrogen (<0.1 wt.%). Meanwhile, the MgH_2_ + 10 wt.% CoTiO_3_ sample desorbed at approximately 2.3 wt.% H_2_, which represents a 23-times-faster desorption rate when compared with undoped MgH_2_. In the same period, the 5, 15, and 20 wt.%-doped MgH_2_ desorbed 0.8, 1.8, and 1.2 wt.% H_2_, respectively, which is also faster than undoped MgH_2_. Table 1 summarizes the hydrogen storage behavior of undoped and doped MgH_2_ with a difference percentage of CoTiO_3_. Notably, low catalyst content is also beneficial in ameliorating the kinetics performances of MgH_2_. Similar to the TPD result, the MgH_2_ + 10 wt.% CoTiO_3_ performs the fastest absorption and desorption rate. These findings are in line with a previous study that demonstrated MgH_2_ + 0.5 mol Nb_2_O_5_ performs superior desorption kinetics than MgH_2_ doped with 0.05, 0.1, 0.2, and 1.0 mol Nb_2_O_5_ [55]. Similar to a study conducted by Ranjbar et al. [54], the desorption behavior of the MgH_2_ with 10 wt.% of SiC presents a faster desorption rate, which becomes slower when doping with 20 wt.% of SiC. They indicated that excessive catalysts in the composite may restrict the hydrogen diffusion to some extent, limiting the Mg–H reaction. From the findings, noticeably, the performance of MgH_2_ can be affected by the amount of the catalyst. By comprehensively considering the absorption/desorption hydrogen performance, the MgH_2_ with 10 wt.% of the CoTiO_3_ sample demonstrates the ultimate absorption/desorption performance. Thus, in this study, the MgH_2_ + 10 wt.% CoTiO_3_ sample is applied to examine the catalytic activity of CoTiO_3_ on the hydrogen storage behavior of MgH_2_. These results are similar to previous studies, in which 10 wt.% was an optimum amount of additive to provide a synergetic catalytic effect on the hydrogen storage performance of a metal hydride and a complex hydride [56,57]. Moreover, an excessive amount of additive may limit the diffusion of hydrogen to some extent, thereby reducing the reaction between the Mg and the hydrogen [54].

**Figure 3:**
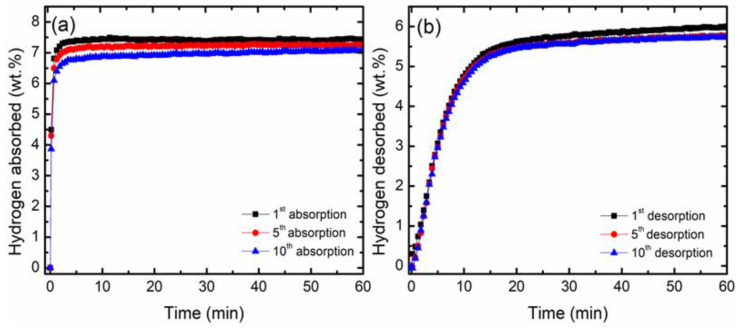
Cycling performance of MgH_2_ + 10 wt.% CoTiO_3_ for (**a**) absorption kinetics curve at 320 °C under 33.0 atm H_2_ pressure and (**b**) desorption kinetics curve at 320 °C under 1.0 atm H_2_ pressure.

The effect of CoTiO_3_ was further explored with the cycling performance of MgH_2_ + 10 wt.% CoTiO_3_, as shown in Figure 3. As reported previously, the cyclability of the undoped MgH_2_ is poor, where the performance would erode drastically after undergoing the first cycle [58]. Therefore, the cyclability of the undoped MgH_2_ is not included in this study because it desorbs only a small amount of hydrogen for the first cycle. Figure 3a shows the absorption kinetics curve of MgH_2_ + 10 wt.% CoTiO_3_ over the ten cycles. Surprisingly, after completing the 10th cycle, there is only a small amount of degradation in the hydrogen capacity for the absorption kinetics performance. The MgH_2_ + 10 wt.% CoTiO_3_ was able to absorb 7.1 wt.% H_2_ after the 10th cycle. Figure 3b shows the desorption kinetics curve of the MgH_2_ + 10 wt.% CoTiO_3_ for the ten cycles. The MgH_2_ + 10 wt.% CoTiO_3_ composite maintain good cyclability, with the ability to desorb 5.7 wt.% H_2_, even after completing the 10th cycle. It is evident that the addition of CoTiO_3_ is beneficial in maintaining a superior cyclability of MgH_2_.

To explore the kinetics mechanism of the MgH_2_ + 10 wt.% CoTiO_3_ composite further, the sorption kinetics performance was analyzed using the kinetics model. For instance, a previous study has demonstrated the kinetic model characterization for the absorption/desorption time for the alloys materials [59,60]. A series of kinetics models have been widely explored and reviewed by Pang and Li [61] in their study. In this study, the kinetics mechanism was studied using Johnson–Mehl–Avrami (JMA) and contracting volume (CV) models, as indicated in Table 2. The JMA and CV models were considered because they are suitable for the experimental data and are precise without requiring further assumptions or approximations [61]. Moreover, other researchers have utilized this method extensively in prior studies to understand the rate-limiting step of the researched materials [62]. With kinetic equations such as CV and JMA, the rate-limiting step of the kinetics can be derived from the experimental data. In this study, the rate-limiting step is determined using the best linear plot of the experimental data with the kinetics equations.

**Figure 4:**
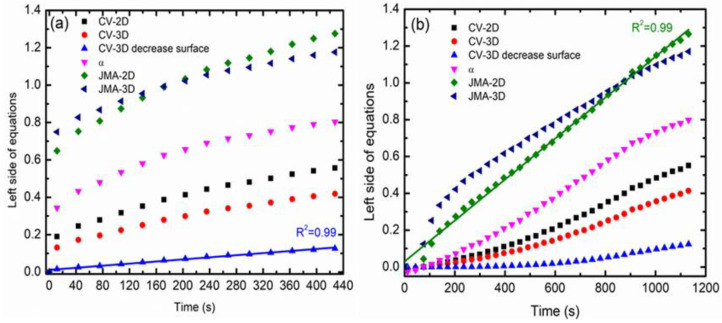
Plot of the resulting curves of different kinetics equations applied to experimental (**a**) absorption kinetics at 200 °C and (**b**) desorption kinetics at 300 °C of MgH_2_ + 10 wt.% CoTiO_3_.

Figure 4 present the kinetics curve that was calculated on the basis of the equation in Table 2. The absorption and desorption kinetics curves of the MgH_2_ + 10 wt.% CoTiO_3_ were calculated for reacted fractions ranging from 0% to 80% [63]. From the figure, for the absorption kinetics at 200 °C, the rate-limiting step is best represented using the 3D growth diffusion controlled with decreasing interface velocity, whereas the desorption kinetics of the MgH_2_ + 10 wt.% CoTiO_3_ at 300 °C was best described using the 2D growth of existing nuclei with constant interface velocity. These results suggest that hydrogen diffusion through the MgH_2_ + 10 wt.% CoTiO_3_ was fast. The nanosized CoTiO_3_ may be beneficial in allowing a faster dissociation rate of hydrogen and hence fasten the diffusion of hydrogen, which results in faster kinetic performance.

**Figure 5:**
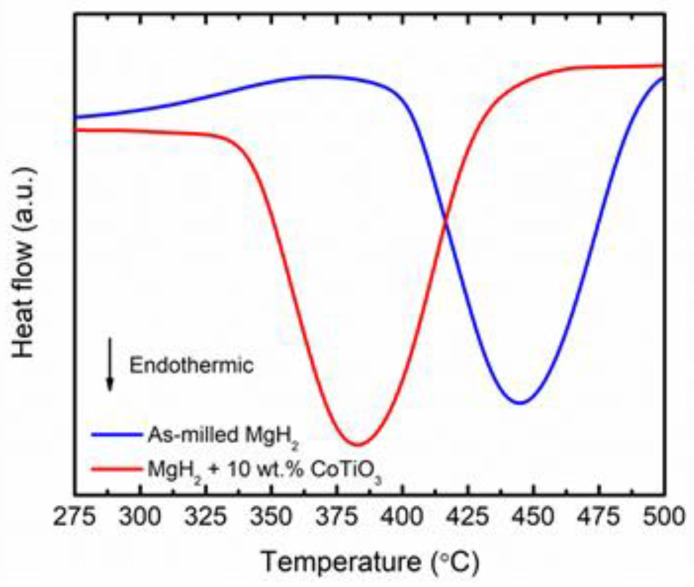
Thermal event of as-milled MgH_2_ and MgH_2_ + 10 wt.% CoTiO_3_ (Heating rate: 30 °C/min) under 50 mL/min of Argon flow.

The thermal characteristics of MgH_2_ + 10 wt.% CoTiO_3_, performed using DSC (heating rate: 30 °C/min), are presented in Figure 5. The DSC trace demonstrates one endothermic peak correlated to the hydrogen release of MgH_2_. The peak of hydrogen release of MgH_2_ + 10 wt.% CoTiO_3_ happens at a lower temperature (382 °C) compared with as-milled MgH_2_ (445 °C), indicating that the dehydrogenation kinetics of MgH_2_ was enhanced by milling with CoTiO_3_.

**Figure 6:**
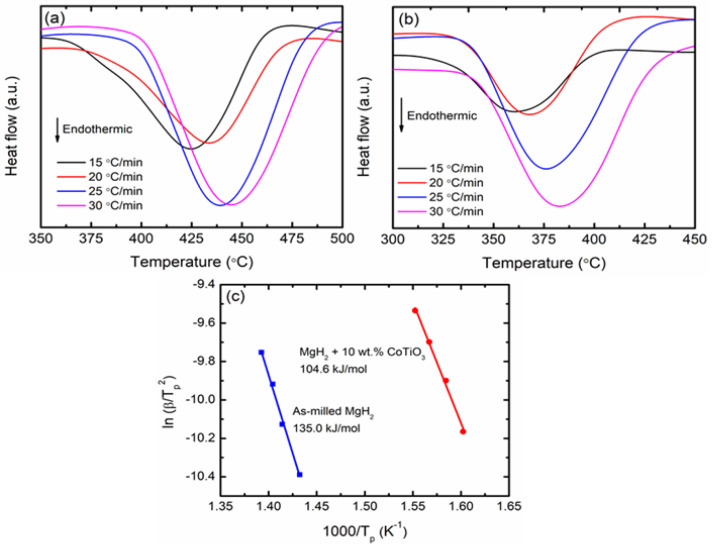
DSC curve of (**a**) as-milled MgH_2_, (**b**) MgH_2_ + 10 wt.% CoTiO_3_ (Heating rate: 15, 20, 25, and 30 °C/min) and (**c**) Kissinger plot of undoped MgH_2_ and MgH_2_ + 10 wt.% CoTiO_3_.

The enhancement in the dehydrogenation kinetic is associated with the energy barrier for hydrogen released from MgH_2_. In this study, the activation energy for hydrogen release from the doped and undoped MgH_2_ was evaluated using the DSC results. Figure 6a,b display the DSC curve of as-milled MgH_2_ and MgH_2_ + 10 wt.% CoTiO_3_ at four different heating rates. The Kissinger equation (Equation (2)) is used to evaluate the activation energy during decomposition.

ln [β/T_p_^2^] = −E_A_/RT_p_ + A (2)

where E_A_ is the activation energy, R is the gas constant, T_p_ is the endothermic peak corresponding to the decomposition temperature, β is the heating rate, and A is a linear constant. Thereafter, the activation energy is determined on the basis of the Kissinger plot of ln [β/T_p_^2^] versus 1000/T_p_, as in Figure 6c. The activation energy of the MgH_2_ + 10 wt.% CoTiO_3_ and as-milled MgH_2_ were calculated to be 104.6 and 135.0 kJ/mol, respectively. The activation energy of MgH_2_ was decreased by 23% with the presence of CoTiO_3_ compared with undoped MgH_2_. Adding CoTiO_3_ into MgH_2_ remarkably reduces the energy barrier, which led to reduced activation energy and enhanced kinetic performance.

Lower activation energy provided the reduction in the desorption barrier, which results in a dramatic decrease in the decomposition temperature. Low decomposition temperature indicates the weakening of the hydride stability [64]. As the bond energy of the Mg–H is the decisive factor in hydride stability, the reduction in hydride stability is attributed to the bond weakening between Mg and H. Typically, the addition of transition metal elements or their oxides helps in decreasing the binding energy between the Mg and H due to the electron exchange process between the MgH_2_ and additives in oxidation or reduction reactions [65]. As reported by a previous study, adding a catalyst is the most favorable approach for promoting the dissociation of the Mg–H bond by lowering its bond energy, which then offers the reduction of the MgH_2_ stability and decomposition temperature [66]. Thus, it is reasonable to conclude that the addition of CoTiO_3_ is beneficial in promoting the superior performance of MgH_2_ by lowering the energy barrier.

**Figure 7:**
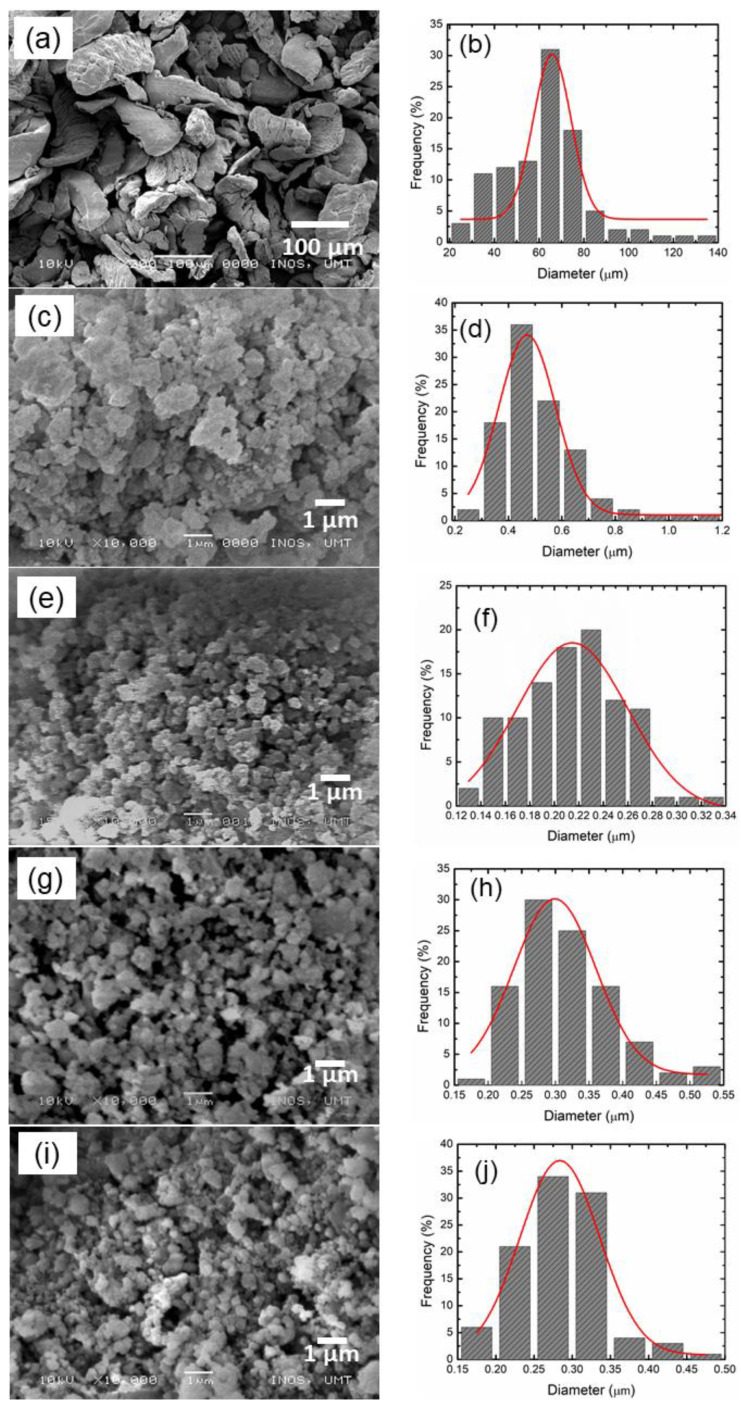
SEM images and particle size distribution histogram of commercial MgH_2_ (**a**,**b**), as-milled MgH_2_ (**c**,**d**), MgH_2_ + 10 wt.% CoTiO_3_ after milling (**e**,**f**), MgH_2_ + 10 wt.% CoTiO_3_ after absorption at 200 °C (**g**,**h**), and MgH_2_ + 10 wt.% CoTiO_3_ after desorption at 450 °C (**i**,**j**).

The catalytic activity of CoTiO_3_ on the microstructure of MgH_2_ was further explored by SEM characterization. Figure 7 displays the micrograph of commercial MgH_2_, MgH_2_ after milling for 1 h, and MgH_2_ + 10 wt.% CoTiO_3_. Referring to Figure 7a, commercial MgH_2_ are irregular in shape, with solid flake-like shaped particles. After being milled for 1 h (Figure 7c), the solid flake-like shaped particles were broken into smaller particles with some agglomerations. Before the milling process, the comparatively smooth surface of the particle is substituted by the asperities and surface defects. Because of these transformed surface structures, as-milled MgH_2_ decomposes at a lower temperature than commercial MgH_2_. Meanwhile, upon the addition of CoTiO_3_ (Figure 7e), the agglomeration was reduced, and the particle size was transformed to a finer shape and size. The size of the MgH_2_ + 10 wt.% CoTiO_3_ was much smaller than ball milled MgH_2_ due to the hardness of CoTiO_3_, which is higher than MgH_2_. The hardness of the MgH_2_ was 0.58 GPa [67], while the hardness of CoTiO_3_ was 3.4–7.5 GPa [68]. Therefore, it is speculated that the CoTiO_3_ introduced the pulverization effect and helped to reduce the particle size of MgH_2_. The particle size of MgH_2_ + 10 wt.% CoTiO_3_ is smaller, and they are finer in shape, even after undergoing the absorption (Figure 7g) and desorption (Figure 7i) process. No big changes occur on the particle after undergoing the absorption and desorption process. From the morphological properties, the CoTiO_3_ is able to reduce the particle size of MgH_2_ and enhances the absorption/desorption performance of MgH_2_. The differences of the microstructure and morphologies of the undoped and doped MgH_2_ may correspond to the presence of a catalyst that is finely dispersed on the surface of the MgH_2_ particle, thus reducing the H diffusion distance and offering more reaction site and hence stimulating the faster absorption and desorption performance of MgH_2_ [69]. This catalyst is embedded on the surface of MgH_2_ and prevent the sample from agglomerating, indicating that CoTiO_3_ is beneficial in distributing particles and constraining agglomeration and particle growth in the MgH_2_ system [22].

The particle size of the commercial MgH_2_, as-milled MgH_2_, MgH_2_ + 10 wt.% CoTiO_3_ after milling as well as after absorption and desorption were further evaluated by Image J software. Figure 7b,d,f,h,j shows the distributions of the particle size, which are depicted in a histogram. Based on the histogram, the average particle size of commercial MgH_2_, as-milled MgH_2_, MgH_2_ + 10 wt.% CoTiO_3_ after milling, after absorption, and after desorption was calculated to be ~60, 0.47, 0.22, 0.29, and 0.28 µm, respectively. The morphological change and drastic size reduction contribute to the high surface defects and more grain boundaries around the surface of the composite [70]. Consequently, a higher amount of reaction for the nucleation sites and better diffusion channels for the hydrogen can be achieved with amplified grain boundaries that enhance the hydrogen sorption performances of the MgH_2_–CoTiO_3_ composite. Similarly, a recent study found that reducing particle size improves absorption–desorption kinetics performance substantially [71,72].

**Figure 8:**
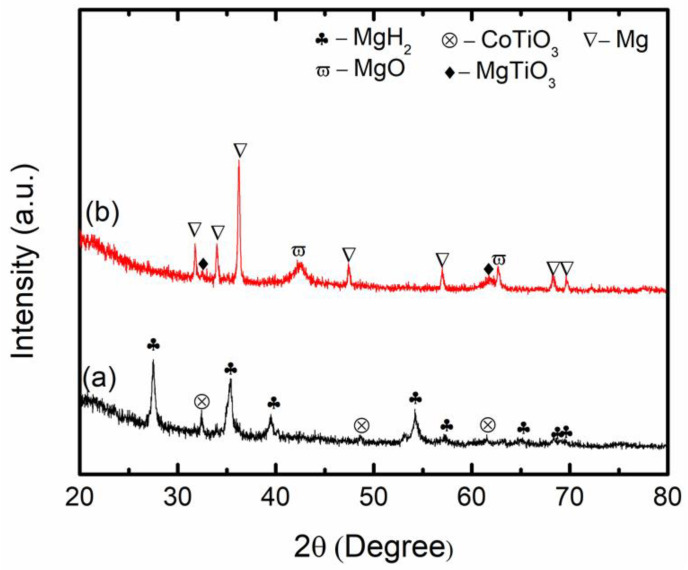
XRD patterns of (**a**) as-milled and (**b**) after desorption at 450 °C of MgH_2_ + 10 wt.% CoTiO_3_.

To reveal the catalytic mechanism of CoTiO_3_ for MgH_2_, XRD analysis for MgH_2_ + 10 wt.% CoTiO_3_ was performed, as in Figure 8. The main peaks that existed after 1 h of milling (Figure 8a) were only MgH_2_ and CoTiO_3_. No new phase was detected, indicating that no chemical reaction occurs during the milling process. Figure 8b presents the sample of the MgH_2_ + CoTiO_3_ after desorption at 450 °C. Peaks of Mg were dominant, indicating that MgH_2_ has been totally transformed to Mg, corresponding to the complete decomposition of MgH_2_, as in (Equation (3)). Additionally, the peaks of MgO and MgTiO_3_ could be detected after dehydrogenation.

MgH_2_ ↔ Mg + H_2_ (3)

**Figure 9:**
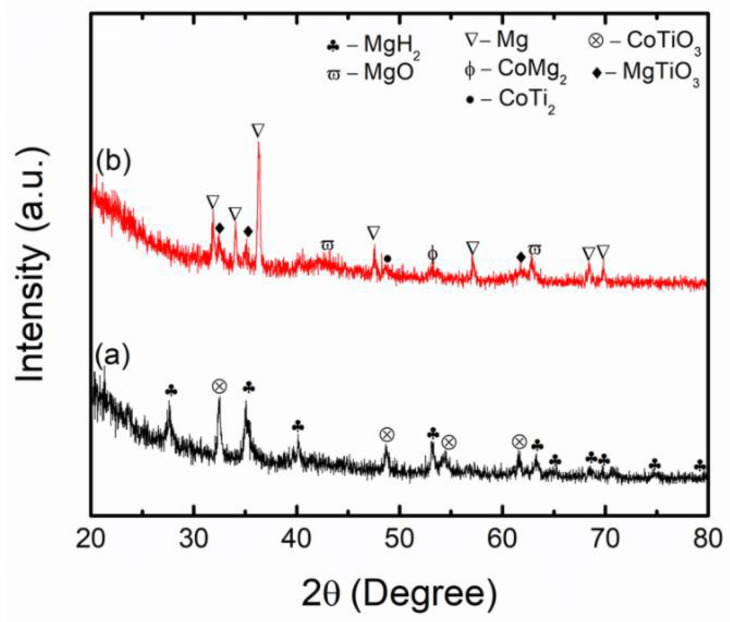
XRD patterns of (**a**) as-milled and (**b**) after desorption at 450 °C of MgH_2_ + 50 wt.% CoTiO_3_.

To further explore the catalytic mechanism of CoTiO_3_ during desorption, a sample of MgH_2_ + 50 wt.% CoTiO_3_ was prepared. As displayed in Figure 9a, after 1 h of milling, only the parent materials, MgH_2_ and CoTiO_3_, were detected. No new compound was formed at this stage. After the desorption process at 450 °C (Figure 9b), the peaks of MgH_2_ and CoTiO_3_ disappeared and new peaks of MgO, MgTiO_3_, CoMg_2_, and CoTi_2_ appeared.

**Figure 10:**
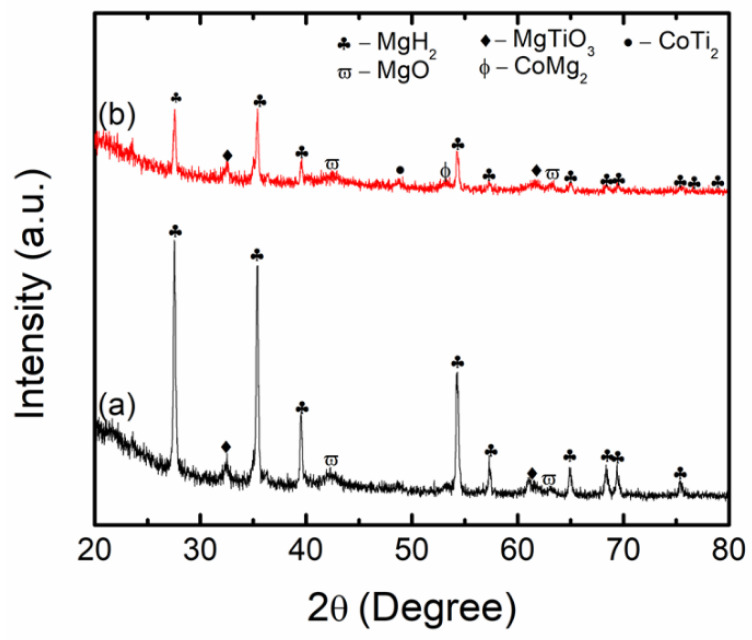
XRD patterns of (**a**) MgH_2_ + 10 wt.% CoTiO_3_ and (**b**) MgH_2_ + 50 wt.% CoTiO_3_ after the absorption process at 200 °C.

**Figure 11:**
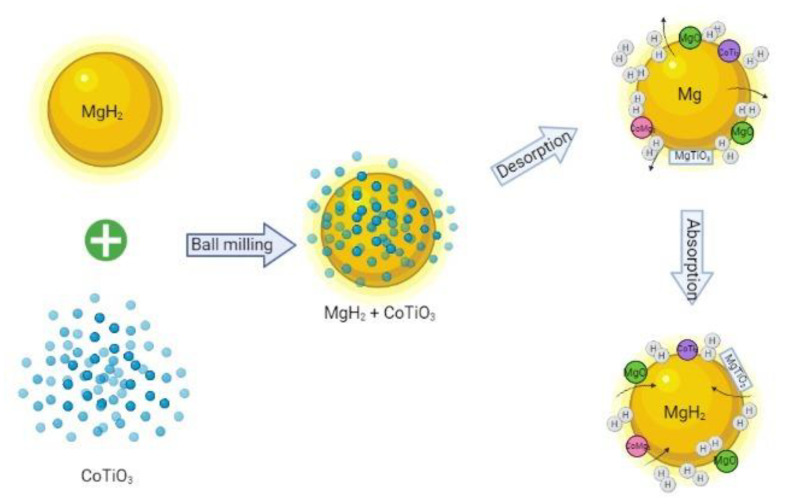
Schematic diagram of the catalytic mechanism of CoTiO_3_ during the absorption/desorption process of the MgH_2_–CoTiO_3_ system. During the milling process, the MgH_2_ matrix reacts with CoTiO_3_. After undergoing the absorption and desorption process, MgTiO_3_, MgO and CoTi_2_, and CoMg_2_ are formed in situ. These in situ active species may serve as an intermediate transfer getaway that accelerates the hydrogen absorption and desorption of MgH_2_. With the assistance of in situ formed MgTiO_3_, MgO and CoTi_2_, and CoMg_2_, the hydrogen storage behavior of MgH_2_ is significantly improved.

Figure 10 presents the MgH_2_ + 10 wt.% CoTiO_3_ and MgH_2_ + 50 wt.% CoTiO_3_ sample after absorption at 200 °C. For the MgH_2_ + 10 wt.% CoTiO_3_ sample (Figure 10a), the main peaks of MgH_2_ were discovered, demonstrating that the Mg was fully converted to MgH_2_, as per Equation (3). The peaks of MgO and MgTiO_3_ were still detected. The appearance of the MgO and MgTiO_3_ species occurred, indicating that MgH_2_ reacts with CoTiO_3_ throughout the heating process. For the MgH_2_ + 50 wt.% CoTiO_3_ sample, similar peaks are presented in the absorption sample (Figure 10b), which are the Mg peaks superseded by the MgH_2_. New peaks of MgO, MgTiO_3_, CoMg_2_, and CoTi_2_ were also detected, suggesting that interaction between MgH_2_ and CoTiO_3_ may occur during the heating process, as indicated in (Equation (4)). The mechanism of the absorption/desorption process of the MgH_2_–CoTiO_3_ system is shown in Figure 11.

11MgH_2_ + 3CoTiO_3_ → 6MgO + MgTiO_3_ + 2CoMg_2_ + CoTi_2_ + 11H_2_ (4)

On the basis of the above result, we postulate that the MgH_2_–CoTiO_3_ system presents a superior hydrogen storage performance of MgH_2_ due to the synergetic effect of the in situ formation of MgTiO_3_, CoMg_2_, CoTi_2_, and MgO. These active species act like active sites at the surface of the MgH_2_ matrix and provide a fast channel for the diffusion of H atoms in the absorption and desorption process [73,74]. For instance, a previous study stated that adding MgTiO_3_ ameliorates the sorption kinetics performance of MgH_2_ [75]. The MgH_2_ + MgTiO_3_ absorbed 5 wt.% of H_2_ in 500 s at 307 °C. Gao et al. [69] also presented the notable performance of MgH_2_ with the formation of Co-containing material after the desorption and absorption processes. It is believed that 3D electron orbitals in the transition metal stimulate the dissociation of H_2_ molecules by serving as the antibonding of H_2_ molecules. Thereafter, Ares-Fernandez and Aguey-Zinsou [50] presented faster absorption/desorption rates of MgH_2_ when it was doped with MgO. In another work, they stated that MgO may take part as a process control agent that can reduce and prevent MgH_2_ from agglomerating by achieving an optimal breakage rate [76]. The catalytic effect of MgTiO_3_, CoMg_2_, CoTi_2_, and MgO could work together to provide a synergetic effect and further boost the performance of MgH_2_. Nevertheless, more research is needed, such as applying X-ray photoelectron spectroscopy and high-resolution transmission electron microscopy, to elucidate the catalytic function of CoTiO_3_ on the hydrogen storage properties of MgH_2_.

## 4. Conclusions

The hydrogen storage performance of MgH_2_ was enhanced with the addition of nanosized CoTiO_3_, which was synthesized via the solid-state method. The different weight percentage of CoTiO_3_ was added into MgH_2_, and the 10 wt.% of the CoTiO_3_-doped MgH_2_ sample present a superior performance of hydrogen storage properties. The MgH_2_ + 10 wt.% CoTiO_3_ started releasing hydrogen at a temperature of 275 °C, 65 °C lower than as-milled MgH_2_ (340 °C). The MgH_2_ + 10 wt.% CoTiO_3_ composite also presents a faster absorption and desorption rate when it can absorb 6.4 wt.% H_2_ in the first 10 min and performs a 23-times-faster desorption rate when compared with as-milled MgH_2_. Moreover, the activation energy of the MgH_2_ decreased from 135 kJ/mol to 104.6 kJ/mol after the addition of 10 wt.% of CoTiO_3_. Adding nanosized CoTiO_3_ also results in a smaller and fine particle that promotes the favorable improvement of MgH_2_ performance. The superior performance of MgH_2_ with the addition of CoTiO_3_ was also attributed to the in situ formation of MgTiO_3_, CoMg_2_, CoTi_2_, and MgO during the heating process, which offers a synergetic catalytic role in improving the performance of MgH_2_. These findings may be beneficial for the modification of the MgH_2_ system for solid-state hydrogen storage in the future.

## Figures and Tables

**Table 1:** The hydrogen storage behavior of undoped and doped MgH_2_ with different weight percentage of CoTiO_3_.

Sample	Decomposition Temperature (°C)	Absorption Capacity (wt.%) in 10 min	Desorption Capacity (wt.%) in 10 min
Commercial MgH_2_	420	-	-
As-milled MgH_2_	340	4.3	< 0.1
MgH_2_ + 5 wt.% CoTiO_3_	298	2.5	0.8
MgH_2_ + 10 wt.% CoTiO_3_	275	6.4	2.3
MgH_2_ + 15 wt.% CoTiO3	276	4.1	1.8
MgH_2_ + 20 wt.% CoTiO_3_	295	6.0	1.2

**Table 2:** Kinetic model used for sorption kinetics of current study, where α, t, and k refer to reacted fraction, time, and reaction rate constant, respectively. Based on [62].

Kinetic Equation	Rate Limiting Step
α = kt	Surface controlled.
1-(1-α)1/3 = kt	CV three-dimensional (3D): contracting volume, 3D growth with constant interface velocity.
1-(1-α)1/2 = kt	CV two-dimensional (2D): contracting volume, 2D growth with constant interface velocity.
1-(2α/3)-(1-α)2/3 = kt	CV 3D (variable velocity): contracting volume, 3D growth diffusion controlled with decreasing interface velocity.
[-ln(1-α)]1/3 = kt	JMA 3D: 3D growth of existing nuclei with constant interface velocity.
[-ln(1-α)]1/2 = kt	JMA 2D: 2D growth of existing nuclei with constant interface velocity.

## Data Availability

The data presented in this study are available on request from the corresponding author.
